# Characterizing differences in retinal and choroidal microvasculature and structure in individuals with Huntington’s Disease compared to healthy controls: A cross-sectional prospective study

**DOI:** 10.1371/journal.pone.0296742

**Published:** 2024-01-30

**Authors:** Suzanna Joseph, Cason B. Robbins, Alice Haystead, Angela Hemesath, Ariana Allen, Anita Kundu, Justin P. Ma, Burton L. Scott, Kathryn P. L. Moore, Rupesh Agrawal, Vithiya Gunasan, Sandra S. Stinnett, Dilraj S. Grewal, Sharon Fekrat

**Affiliations:** 1 Department of Ophthalmology, Duke University School of Medicine, Durham, NC, United States of America; 2 iMIND Research Group, Durham, NC, United States of America; 3 Department of Neurology, Duke University School of Medicine, Durham, NC, United States of America; 4 National Healthcare Group Eye Institute, Tan Tock Seng Hospital, Singapore, Singapore; 5 Lee Kong Chian School of Medicine, Nanyang Technological University, Singapore, Singapore; University of Catania Department of Surgical and Medical Sciences Advanced Technologies GF Ingrassia: Universita degli Studi di Catania Dipartimento di Scienze Mediche Chirurgiche e Tecnologie Avanzate GF Ingrassia, ITALY

## Abstract

**Objective:**

To characterize retinal and choroidal microvascular and structural changes in patients who are gene positive for mutant huntingtin protein (mHtt) with symptoms of Huntington’s Disease (HD).

**Methods:**

This study is a cross-sectional comparison of patients who are gene positive for mHtt and exhibit symptoms of HD, either motor manifest or prodromal (HD group), and cognitively normal individuals without a family history of HD (control group). HD patients were diagnosed by Duke movement disorder neurologists based on the Unified Huntington’s Disease Rating Scale (UHDRS). Fovea and optic nerve centered OCT and OCTA images were captured using Zeiss Cirrus HD-5000 with AngioPlex. Outcome metrics included central subfield thickness (CST), peripapillary retinal nerve fiber layer (pRNFL) thickness, ganglion cell-inner plexiform layer (GCIPL) thickness, and choroidal vascularity index (CVI) on OCT, and foveal avascular zone (FAZ) area, vessel density (VD), perfusion density (PD), capillary perfusion density (CPD), and capillary flux index (CFI) on OCTA. Generalized estimating equation (GEE) models were used to account for inter-eye correlation.

**Results:**

Forty-four eyes of 23 patients in the HD group and 77 eyes of 39 patients in the control group were analyzed. Average GCIPL thickness and FAZ area were decreased in the HD group compared to controls (p = 0.001, p < 0.001). No other imaging metrics were significantly different between groups.

**Conclusions:**

Patients in the HD group had decreased GCIPL thickness and smaller FAZ area, highlighting the potential use of retinal biomarkers in detecting neurodegenerative changes in HD.

## Introduction

Huntington’s Disease (HD) is a neurodegenerative movement disorder characterized by motor, cognitive, and psychiatric symptoms [1]. As a monogenic disease, HD is caused by an autosomal dominant trinucleotide repeat of CAG (cytosine-adenine-guanine) in chromosome 4, leading to the production of a mutant huntingtin protein (mHtt) [2]. This unique pathophysiology allows for predictive genetic testing; however, it is limited in its ability to evaluate disease progression and severity [3]. Treatment for HD is primarily symptomatic, with no current disease-modifying therapy available [1]. With several clinical trials underway seeking to identify a disease-modifying treatment, there has been increased interest in identifying HD biomarkers to monitor both disease progression and treatment response [1, 3–5].

A growing body of literature has suggested potential retinal biomarkers for various neurodegenerative diseases [6–9]. The retina and the brain are developmentally derived from the same tissue and, as such, share the same microvascular structure [8]. However, in contrast to the brain, the unique anatomical structure of the eye lends itself well to optical imaging that is both high resolution and non-invasive, making it an ideal surrogate for monitoring changes in central nervous system architecture [8]. However, little is known about retinal manifestations in eyes of individuals with HD. Prior studies have found attenuation of various structural layers including the peripapillary retinal nerve fiber layer (pRNFL), ganglion cell layer, and subfoveal choroid [10, 11]. However, changes in choroidal vascularity index (CVI) and retinal microvascular density are, as of yet, uncharacterized. In this study, we used optical coherence tomography (OCT) and OCT angiography (OCTA) to characterize retinal and choroidal microvascular and structural changes in eyes of individuals who are gene-positive for mHtt and exhibit symptoms suggestive of HD compared to controls with normal cognition and no family history of HD.

## Materials and methods

This prospective cross-sectional study was approved by the Duke Health Institutional Review Board (Pro00082598). It was in compliance with the Health Insurance Portability and Accountability Act of 1996 and abided by all tenets of the Declaration of Helsinki. Prior to study enrollment, written informed consent was obtained by all subjects or their legally authorized representative. Participants were recruited from January 2019 to January 2023. Study size included all patients who were able to be enrolled during this time period.

### Study participants

Prospective study participants were individuals who were gene-positive for mHtt (≥ 36 CAG repeats) and exhibited clinical symptoms suggestive of HD. The HD group consisted of patients with either motor manifest HD or prodromal HD. Patients were seen at Duke Neurological Disorders Clinic and diagnosed by Duke movement disorder neurologists (BLS and KPLM) based on genetic screening and the Unified Huntington’s Disease Rating Scale (UHDRS). Motor manifest HD was defined as genetically positive patients with UHDRS motor diagnostic confidence score of 4, indicating “motor abnormalities that are unequivocal signs of HD (>99% confidence interval)” [12]. Prodromal HD was defined as genetically positive patients with non-specific motor, cognitive, or behavioral signs as defined by the movement disorder society [13, 14]. Prodromal HD participants had UHDRS motor diagnostic confidence score of 1–3 indicating motor abnormalities that range from “nonspecific for HD” to “likely signs of HD” [12]. The words “prodromal” or “premanifest” were explicitly stated in participant’s medical records. Gene expansion repeat numbers were recorded for all participants in the HD group.

The control group consisted of individuals with normal cognition and no family history of HD. This was done to limit confounding variables from participants with possible undiagnosed dementia or memory decline as prior work has reported retinal changes in individuals with various neurodegenerative conditions [6–9]. Control participants were not tested for mHtt, given the autosomal dominant inheritance and manifestation of the disease. Participants were recruited from Duke Neurological Disorders Clinic, the surrounding community, and the Duke Alzheimer’s Disease Prevention Registry of research volunteers with normal cognition. Participants from the Duke Alzheimer’s Disease Prevention Registry underwent an extensive battery of neuropsychological testing including the Montreal Cognitive Assessment (MoCA, score of 26 or greater) to confirm normal cognition.

Participants underwent measurement of corrected visual acuity on a Snellen chart, a cognitive evaluation with Mini-Mental State Examination (MMSE), and a brief questionnaire including family history of neurodegenerative disorders and years of education from the first grade onwards. Participants and their caregivers were also asked about previous history of ophthalmic conditions at time of imaging which was then confirmed through medical record review when available. Authors had access to information that could identify individual participants during data collection. Any fundus pathology in participants was screened for through nonmydriatic ultra-widefield scanning laser ophthalmoscopy imaging (Optos California, Optos, Marlborough, MA) and if present, those individuals were excluded. Other exclusion criteria included history of diabetes mellitus, glaucoma, uncontrolled hypertension, other neurodegenerative disease, Snellen visual acuity worse than 20/40 at the time of data collection, and spherical equivalent (SEQ) of less than -6 diopters (D) or greater than +6D.

### OCTA and OCT image acquisition and protocols

The Zeiss Cirrus HD-OCT 5000 with AngioPlex OCTA (Software Version 11.0.0.29946; Carl Zeiss Meditec, Dublin, CA) was used to image all patients [15]. Both 3x3mm and 6x6mm OCTA scans centered on the fovea were taken. Superficial capillary plexus (SCP) vessel density (VD) and perfusion density (PD) were measured in the Early Treatment Diabetic Retinopathy Study (ETDRS) grid overlay using the 3x3mm ETDRS ring and circle and the 6x6mm ETDRS inner and outer rings and circle. VD was defined as the total length of perfused vasculature over the area of interest while PD was defined as the percentage of perfused vasculature area over the area of interest. Foveal avascular zone (FAZ) boundaries in the SCP were automatically detected and the total area was recorded. Study staff manually reviewed all automatically detected FAZ boundaries and adjusted for any inaccuracies. To assess the radial peripapillary capillary (RPC) plexus, specifically capillary perfusion density (CPD) and capillary flux index (CFI), 4.5x4.5mm images centered on the optic nerve head were obtained. CPD and CFI were calculated using a vessel skeleton map. The vessel skeleton map was created using a thresholding algorithm which linearized a binary vessel slab into one-pixel widths. CPD was defined as the percentage of perfused capillary vasculature over area of interest. CFI was defined as a unitless ratio of perfused capillary vasculature weighted by normalized flow intensity over area of interest. Vessel pixel brightness in the *en face* image was used to quantify flow intensity [16]. CFI represents the proportion of red blood cells passing through a given capillary bed at a given point in time.

OCT images including 512 x128μm macular cube, 200x200μm optic disc cube, and a 21-line raster scan of the posterior pole (high-definition-21 line foveal image) with enhanced depth imaging (EDI) were acquired. The central subfield thickness (CST) and average ganglion cell inner plexiform layer (GCIPL) thickness were calculated using the 512x128μm macular cube image. CST was defined as the distance between the inner limiting membrane at the fovea and the retinal pigment epithelium. A 14.13mm^2^ elliptical centered on the fovea was used to calculate the average GCIPL thickness. A 3.46-mm diameter circle was centered on the optic disc of the 200x200μm optic disc cube scan to calculate the pRNFL thickness.

Choroidal vascularity index (CVI), a measurement of the choroid’s vascularity, was calculated using the Comprehensive Ocular Imaging Network which analyzed high-definition 21-line foveal images with EDI. Methods have been described by Agrawal et al. [17]. In brief, the image was binarized using ImageJ, version 1.52r26 (National Institutes of Health). A polygonal tool was used to select the total subfoveal choroidal area (TCA). This was then used to create a 1.5mm segment of the subfoveal choroidal area. A color threshold tool was used to select the dark pixels of the binarized image, which was defined as the luminal area (LA). CVI was calculated by dividing LA by TCA [18].

Trained study staff manually assessed the quality of each image at the time of data collection based on the OSCAR-IB criteria [19]. Image quality was subsequently reviewed by masked study staff prior to data analysis. Those images with poor scan quality (less than 7/10 signal strength index or significant image artifact) were excluded from statistical analysis.

### Data analysis

At the time of data analysis all collected information was deidentified. Imaging metrics from the HD group were compared to the control group. SAS/STAT software, Version 9.4 of the SAS System for Windows (2002–2012 SAS Institute Inc.) was used to complete all statistical analyses. To assess for possible confounders, demographic characteristics were compared. Fisher’s exact test of differences between proportions and the Wilcoxon rank sum test were used for categorical and continuous variables, respectively. Imaging parameters were compared with generalized estimating equations (GEE) in which age and sex were controlled for as covariates. GEE models were used to account for the correlation between 2 eyes of the same study participant. A Spearman’s rank correlation coefficient analysis was used to assess the relationship between the number of CAG repeats in a patient and their retinal imaging parameters. A Bonferroni correction was used to limit the increased error rate given the multiple comparisons used in the study. As such a p-value of < 0.002 was considered statistically significant.

## Results

A total of 54 eyes from 28 participants in the HD group were imaged. Of these 8 eyes, from 4 participants were excluded because they were asymptomatic for any of the motor, cognitive, or behavioral signs of motor manifest or prodromal HD. Two eyes from 1 patient were excluded due to a diagnosis of diabetes. Two eyes from 2 participants were excluded due to retinal detachment. In the control group, a total of 77 eyes of 39 patients were imaged. Of these, 1 eye from 1 patient was excluded due to a Snellen visual acutity lower than 20/40. Forty-four eyes of 23 participants in the HD group (mean [SD] age, 54.9 [14.6]; 10 men [43.5%]), and 77 eyes of 39 patients in the control group (mean [SD] age, 56.9 [15.0]; 19 men [50.0%]) were analyzed. Of those in the HD group, 36 eyes of 19 patients were motor manifest HD and 8 eyes of 4 patients were prodromal HD. The CAG gene expansion repeats in the HD group ranged from 37 to 49. In the prodromal HD group, the gene expansion repeats ranged from 37 to 44. Table 1 describes demographics of the study population analyzed. Participants in the HD and control groups did not differ in age and sex (p = 0.534, p = 0.792). Patients in the HD group had lower MMSE scores (28.0 [2.4] vs 29.7 [0.7], p < 0.001).

**Table 1:** Demographics.

Variable	Statistic	HD	Controls	P-value*
Age	N	23	39	
	Mean (SD)	54.9 (14.6)	56.9 (15.0)	0.534
	Min, Median, Max	29, 54, 79	27, 59, 77	
MMSE	N	20	39	
	Mean (SD)	28.0 (2.4)	29.7 (0.7)	**<0.001**
	Min, Median, Max	21, 28, 30	27, 30, 30	
Male gender	N (%)	10(43.5)	19 (48.7)	0.792
Smoking history	N (%)	11 (47.8)	6 (15.4)	0.228

*P-values for age and MMSE based on Wilcoxon rank sum test.

P-value for gender and smoking based on Fisher’s exact test.

HD: Huntington’s disease

MMSE: Mini-mental state examination

**Fig 1:**
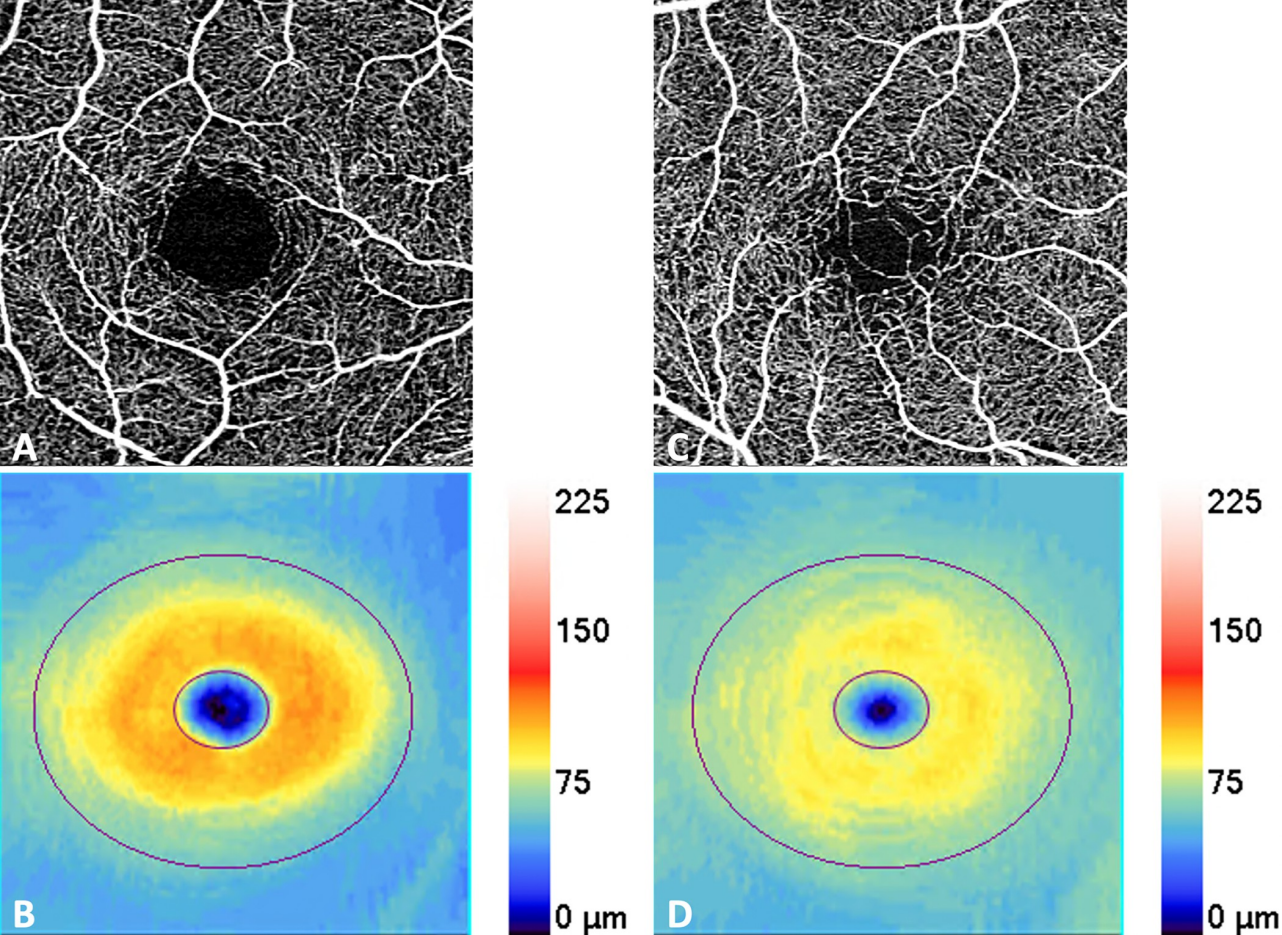
Representative Optical Coherence Tomography Angiography (OCTA) scan of control and Huntington’s Disease participants. Representative OCTA 3x3mm images of the superficial capillary plexus (SCP) of the right eye of a 38 year old male control subject (A) and the right eye of a 44 year old male Huntington’s Disease (HD) subject (C) show decreased FAZ area in the subject with HD. Corresponding quantitative color maps (Carl Zeiss Meditec, Dublin, CA) of the ganglion cell layer, with the scale on the right show attenuation in the subject with HD (D) compared to the control subject (B).

Table 2 shows the results of GEE analysis of OCTA parameters in the HD and control groups. The HD group had decreased FAZ area (0.178mm^2^ [0.064] vs 0.248mm^2^ [0.091], p < 0.001) as seen in Fig 1. The 2 groups did not differ with regard to VD, PD, CPD, or CFI metrics.

**Table 2:** Generalized estimating equation analysis on OCTA parameters in individuals in the HD group and control group.

	Variable	HD	Controls	P-value*
**PERFUSION DENSITY (3X3MM)**	N	36	53	
	Mean (SD)	0.364 (0.030)	0.362 (0.023)	0.695
	Min, Median, Max	0.272, 0.371, 0.413	0.323, 0.364, 0.409	
**PERFUSION DENSITY IN RING (3X3MM)**	N	36	53	
	Mean (SD)	0.382 (0.030)	0.382 (0.022)	0.997
	Min, Median, Max	0.298, 0.387, 0.431	0.334, 0.382, 0.434	
**VESSEL DENSITY (3X3MM)**	N	36	53	
	Mean (SD)	20.3 (1.7)	20.3 (1.2)	0.939
	Min, Median, Max	14.9, 20.7, 23.0	18.1, 20.2, 22.7	
**VESSEL DENSITY IN RING (3X3MM)**	N	36	53	
	Mean (SD)	21.3 (1.7)	21.3 (1.2)	0.716
	Min, Median, Max	16.2, 21.5, 24.0	18.7, 21.4, 23.4	
**PERFUSION DENSITY (6X6MM)**	N	36	35	
	Mean (SD)	0.435 (0.032)	0.436 (0.028)	0.582
	Min, Median, Max	0.320, 0.444, 0.477	0.377, 0.447, 0.479	
**PERFUSION DENSITY INNER RING (6X6MM)**	N	36	35	
	Mean (SD)	0.433 (0.032)	0.429 (0.029)	0.840
	Min, Median, Max	0.314, 0.438, 0.474	0.369, 0.433, 0.473	
**PERFUSION DENSITY OUTER RING (6X6MM)**	N	36	35	
	Mean (SD)	0.444 (0.029)	0.446 (0.029)	0.411
	Min, Median, Max	0.364, 0.451, 0.485	0.386, 0.454, 0.489	
**VESSEL DENSITY (6X6MM)**	N	36	35	
	Mean (SD)	17.8 (1.1)	17.9 (1.1)	0.394
	Min, Median, Max	13.8, 18.2, 19.3	15.8, 18.2, 19.7	
**VESSEL DENSITY INNER RING (6X6MM)**	N	36	35	
	Mean (SD)	18.1 (1.0)	18.0 (1.1)	0.771
	Min, Median, Max	14.6, 18.3, 19.4	15.7, 18.0, 19.8	
**VESSEL DENSITY OUTER RING (6X6MM)**	N	36	35	
	Mean (SD)	18.0 (1.2)	18.2 (1.1)	0.166
	Min, Median, Max	13.5, 18.4, 19.4	16.1, 18.5, 19.9	
**AVERAGE CAPILLARY PERFUSION DENSITY**	N	36	66	
	Mean (SD)	0.443 (0.015)	0.442 (0.018)	0.855
	Min, Median, Max	0.411, 0.441, 0.476	0.379, 0.442, 0.478	
**AVERAGE CAPILLARY FLUX INDEX**	N	36	66	
	Mean (SD)	0.456 (0.019)	0.452 (0.024)	0.786
	Min, Median, Max	0.405, 0.459, 0.480	0.387, 0.456, 0.502	
**AREA OF FOVEAL AVASCULAR ZONE**	N	33	51	
	Mean (SD)	0.178 (0.064)	0.248 (0.091)	**<0.001**
	Min, Median, Max	0.060, 0.180, 0.320	0.040, 0.260, 0.520	

*P-value based comparison of means between groups using generalized estimating equations (GEE) to control for correlation between eyes of same patient and adjusting for age and sex.

OCTA: optical coherence tomography angiography

HD: Huntington’s disease

Table 3 shows the results of the GEE analysis of OCT parameters in the HD and control groups. The HD group had decreased average GCIPL (76.0μm [5.9] vs. 80.1μm [6.3], p = 0.001). The 2 groups did not differ with regard to CST, average pRNFL thickness, quadrant pRNFL thickness, or CVI metrics.

**Table 3:** Generalized estimating equation analysis on OCT parameters in individuals in the HD group and control group.

Variable	Statistic	HD	Controls	P-value*
Central subfield thickness	N	41	75	
	Mean (SD)	259.0 (19.5)	261.9 (20.7)	0.674
	Min, Median, Max	217.0, 260.0, 291.0	227.0, 260.0, 333.0	
Peripapillary retinal nerve fiber layer thickness (pRNFL), μm	N	36	77	
	Mean (SD)	92.0 (10.3)	90.8 (9.8)	0.842
	Min, Median, Max	70.0, 92.0, 113.0	65.0, 91.0, 112.0	
pRNFL thickness—superior quadrant, μm	N	36	77	
	Mean (SD)	111.5 (15.42)	109.6 (13.21)	0.924
	Min, Median, Max	82.0, 113.5, 159.0	80.0, 110.0, 134.0	
pRNFL thickness—nasal quadrant, μm	N	36	77	
	Mean (SD)	71.69 (8.47)	71.99 (12.73)	0.929
	Min, Median, Max	58.0, 72.0, 89.0	45.0, 70.0, 117.0	
pRNFL thickness—inferior quadrant, μm	N	36	77	
	Mean (SD)	121.8 (20.37)	117.1 (18.25)	0.465
	Min, Median, Max	65.0, 126.0, 151.0	56.0, 117.0, 152.0	
pRNFL thickness—temporal quadrant, μm	N	36	77	
	Mean (SD)	63.08 (10.20)	64.52 (10.51)	0.346
	Min, Median, Max	37.0, 64.5, 80.0	41.0, 64.0, 91.0	
Average ganglion cell-inner plexiform layer thickness	N	38	71	
	Mean (SD)	76.0 (5.9)	80.1 (6.3)	**0.001**
	Min, Median, Max	62.0, 77.5, 86.0	57.0, 82.0, 91.0	
CVI	N	35	47	
	Mean (SD)	0.655 (0.018)	0.651 (0.014)	0.419
	Min, Median, Max	0.636, 0.649, 0.722	0.626, 0.651, 0.681	

*P-value based comparison of means between groups using generalized estimating equations (GEE) to control for correlation between eyes of same patient and adjusting for age and sex.

OCT: optical coherence tomography

HD: Huntington’s disease

CVI: choroidal vascularity index

A secondary analysis was performed comparing only motor manifest HD patients (rather than pooled motor manifest and prodromal HD patients as in the primary analysis) to the control group (Tables 4 and 5). The results remained similar, with decreased FAZ area in motor manifest HD patients compared to controls with normal cognition (p < 0.001). However, there was no significant difference seen in GCIPL thickness between the 2 groups (p = 0.004). Additionally, there were no significant differences found in regard to PD, CPD, CFI, CST, or CVI metrics.

**Table 4:** Generalized estimating equation analysis on OCTA parameters in individuals with motor manifest HD and controls.

	Variable	Motor manifest	Controls	P-value*
**PERFUSION DENSITY (3X3MM)**	N	28	53	
	Mean (SD)	0.360 (0.031)	0.362 (0.023)	0.939
	Min, Median, Max	0.272, 0.369, 0.411	0.323, 0.364, 0.409	
**PERFUSION DENSITY IN RING (3X3MM)**	N	28	53	
	Mean (SD)	0.378 (0.031)	0.382 (0.022)	0.573
	Min, Median, Max	0.298, 0.387, 0.429	0.334, 0.382, 0.434	
**VESSEL DENSITY (3X3MM)**	N	28	53	
	Mean (SD)	20.1 (1.8)	20.3 (1.2)	0.575
	Min, Median, Max	14.9, 20.6, 22.2	18.1, 20.2, 22.7	
**VESSEL DENSITY IN RING (3X3MM)**	N	28	53	
	Mean (SD)	21.0 (1.7)	21.3 (1.2)	0.281
	Min, Median, Max	16.2, 21.5, 23.2	18.7, 21.4, 23.4	
**PERFUSION DENSITY (6X6MM)**	N	28	35	
	Mean (SD)	0.429 (0.032)	0.436 (0.028)	0.139
	Min, Median, Max	0.320, 0.441, 0.466	0.377, 0.447, 0.479	
**PERFUSION DENSITY INNER RING (6X6MM)**	N	28	35	
	Mean (SD)	0.426 (0.033)	0.429 (0.029)	0.442
	Min, Median, Max	0.314, 0.432, 0.474	0.369, 0.433, 0.473	
**PERFUSION DENSITY OUTER RING (6X6MM)**	N	28	35	
	Mean (SD)	0.438 (0.030)	0.446 (0.029)	0.082
	Min, Median, Max	0.364, 0.449, 0.479	0.386, 0.454, 0.489	
**VESSEL DENSITY (6X6MM)**	N	28	35	
	Mean (SD)	17.6 (1.1)	17.9 (1.1)	0.060
	Min, Median, Max	13.8, 18.1, 19.0	15.8, 18.2, 19.7	
**VESSEL DENSITY INNER RING (6X6MM)**	N	28	35	
	Mean (SD)	17.9 (1.0)	18.0 (1.1)	0.530
	Min, Median, Max	14.6, 18.1, 19.2	15.7, 18.0, 19.8	
**VESSEL DENSITY OUTER RING (6X6MM)**	N	28	35	
	Mean (SD)	17.8 (1.2)	18.2 (1.1)	0.017
	Min, Median, Max	13.5, 18.1, 19.4	16.1, 18.5, 19.9	
**AVERAGE CAPILLARY PERFUSION DENSITY**	N	28	66	
	Mean (SD)	0.443 (0.017)	0.442 (0.018)	0.708
	Min, Median, Max	0.411, 0.441, 0.476	0.379, 0.442, 0.478	
**AVERAGE CAPILLARY FLUX INDEX**	N	28	66	
	Mean (SD)	0.459 (0.012)	0.452 (0.024)	0.433
	Min, Median, Max	0.423, 0.460, 0.477	0.387, 0.456, 0.502	
**AREA OF FOVEAL AVASCULAR ZONE**	N	25	51	
	Mean (SD)	0.173 (0.066)	0.248 (0.091)	**<0.001**
	Min, Median, Max	0.060, 0.180, 0.320	0.040, 0.260, 0.520	

*P-value based comparison of means between groups using generalized estimating equations (GEE) to control for correlation between eyes of same patient and adjusting for age and sex.

OCTA: optical coherence tomography angiography

HD: Huntington’s disease

**Table 5:** Generalized estimating equation analysis on OCT parameters in individuals with motor manifest HD and controls.

Variable	Statistic	Motor manifest	Controls	P-value*
Central subfield thickness	N	33	75	
	Mean (SD)	258.2 (20.7)	261.9 (20.7)	0.738
	Min, Median, Max	217.0, 260.0, 291.0	227.0, 260.0, 333.0	
Average peripapillary retinal nerve fiber layer thickness (pRNFL), μm	N	28	77	
	Mean (SD)	93.4 (9.7)	90.8 (9.8)	0.611
	Min, Median, Max	75.0, 92.5, 113.0	65.0, 91.0, 112.0	
pRNFL thickness—superior quadrant, μm	N	28	77	
	Mean (SD)	113.0 (14.28)	109.6 (13.21)	0.769
	Min, Median, Max	86.0, 115.0, 159.0	80.0, 110.0, 134.0	
pRNFL thickness—nasal quadrant, μm	N	28	77	
	Mean (SD)	72.07 (9.20)	71.99 (12.73)	0.797
	Min, Median, Max	58.0, 73.5, 89.0	45.0, 70.0, 117.0	
pRNFL thickness—inferior quadrant, μm	N	28	77	
	Mean (SD)	125.8 (16.08)	117.1 (18.25)	0.162
	Min, Median, Max	98.0, 127.5, 151.0	56.0, 117.0, 152.0	
pRNFL thickness—temporal quadrant, μm	N	28	77	
	Mean (SD)	63.00 (11.27)	64.52 (10.51)	0.006
	Min, Median, Max	37.0, 65.5, 80.0	41.0, 64.0, 91.0	
Average ganglion cell-inner plexiform layer thickness	N	30	71	
	Mean (SD)	76.0 (6.5)	80.1 (6.3)	0.004
	Min, Median, Max	62.0, 78.0, 86.0	57.0, 82.0, 91.0	
CVI	N	27	47	
	Mean (SD)	0.653 (0.015)	0.651 (0.014)	0.862
	Min, Median, Max	0.636, 0.647, 0.694	0.626, 0.651, 0.681	

*P-value based comparison of means between groups using generalized estimating equations (GEE) to control for correlation between eyes of same patient and adjusting for age and sex.

OCT: optical coherence tomography

HD: Huntington’s disease

CVI: choroidal vascularity index

A Spearman’s rank correlation coefficient analysis was conducted to assess the relationship between the number of CAG repeats and the measured retinal parameters (S1 Table). The associations between CAG repeats and the analyzed retinal imaging parameters were not statistically significant.

## Discussion

This cross-sectional study is the first to identify decreased FAZ area in the SCP using OCTA in the eyes of individuals positive for mHtt and symptomatic for HD. To the best of our knowledge, only 2 prior studies have used OCTA to assess retinal vascular changes in HD patients [10, 11]. Amini et al. analyzed the macular retinal thickness, peripapillary RNFL thickness, and superficial and deep plexus VD in 46 motor manifest eyes using the SD-OCT by Heidelberg Spectralis (Heidelberg Engineering, Heidelberg, Germany). Maio et al. analyzed the ganglion cell complex, peripapillary RNFL thickness, choriocapillaris thickness, central choroidal thickness, and superficial and deep plexus VD in 32 motor manifest eyes using the Optovue RTVue (Optovue Inc, Freemont, CA) [10]. Both studies accounted for the non-independence of 2 eyes from the same subject. These studies found no differences in VD among motor manifest HD and control participants, aligning with the results found in our study [10, 11]. Our study, however, included a pooled cohort consisting of both motor manifest HD and prodromal HD. This is a novel study that analyzes OCTA images in individuals with prodromal HD. Furthermore, we analyzed FAZ area, PD, CPD, CFI, and CVI which have all been unstudied in prior literature on HD. In addition to the decreased FAZ area, we found significantly attenuated GCIPL thickness in the HD group compared to control group. CVI did not significantly differ between groups. Furthermore, this study found no significant relationship between the number of CAG repeats and retinal parameters assessed.

Our study found decreased FAZ area in the eyes of the HD group compared to controls. The FAZ is an area characterized by the absence of any blood vessels. It overlies the foveal pit, which consists exclusively of photoreceptors, specifically cones, and is considered the anatomic location of sharpest visual acuity in the retinal tissue [20]. Foveal architectural changes are thought to be biomarkers for various pathologic processes [21]. While the mechanism behind decreased FAZ area in HD remains unclear, we hypothesize that it could be related to HD-associated photoreceptor degeneration which has been observed in mice and fruit fly models of HD [22–24]. Batcha et al. used electroretinography to detect a deficit in the cone pathway which was found to occur at the onset of motor disturbance [22]. They additionally observed the loss of essential photoreceptor signaling proteins, cone opsin and transducin, through post-mortem immunocytochemical analysis [22]. The HD-driven loss of the photoreceptor’s G-protein coupled receptor and signal transducer (cone opsin and transducin respectively) [25] could be a potential source of the photoreceptor degeneration/ dysregulation seen in animal models of HD and could lead to attenuation of the foveal pit. Foveal pit degeneration has been associated with decreased FAZ area in both ocular albinism [26–28] and Parkinson’s disease (PD) [25], and thus a similar phenomenon may be occurring in HD eyes. This is not to say that individuals with HD have foveal degeneration but rather that neurodegeneration involving the perifovea may be driving microvascular changes resulting in attenuation of the FAZ. Of note, we did not observe any changes in CST thickness in the HD group. However, prior papers have reported retinal microvasculature changes preceding that of gross layer attenuation, suggesting that this difference may be too subtle to reliably detect on OCT [29–31].

In healthy retinas, decreased FAZ area may correspond to increased foveal thickness [32]. However, there is great variability in FAZ size in healthy retinas, with additional conflicting data on associations between FAZ area and demographic variables such as age and sex [33–35]. In a similar vein, the data on FAZ area in Alzheimer’s disease is mixed with some studies reporting no FAZ area change [36, 37] and others reporting increased FAZ area [38, 39]. Since no prior studies on HD eyes have evaluated FAZ area, the clinical significance of this finding is uncertain.

The attenuated GCIPL thickness found in our study confirms prior work that identified thinning in both the ganglion cell layer and the inner plexiform layer in individuals with symptomatic HD [40]. Interestingly, GCIPL attenuation was not found by Mazur- Michałek et al. and Schmid et al. [41, 42]. Both papers had slightly different cohorts than our study, with Mazur-Michałek et al. studying asymptomatic individuals who were genetically positive for HD and Schmid et al. studying both individuals who were genetically positive and in the asymptomatic or the prodromal disease stage [41, 42]. In contrast, our study focused on individuals who were genetically positive and in the prodromal or motor manifest disease stage, and therefore had a more advanced form of HD than the 2 prior which could account for the differences seen in GCIPL thickness. Of note, our motor manifest subcohort did not show significant attenuation of the GCIPL (p = 0.004) which could be due to a relatively limited sample size. The GCIPL is the location of retinal ganglion cell bodies, the only retinal neurons that directly communicate with the brain [43–45]. It is, therefore, unsurprising that attenuation of this layer has been found to be a strong biomarker for various neurodegenerative diseases [46–49]. Despite the thinning seen in the GCIPL layer in HD eyes, our study found no difference in average or quadrant pRNFL thickness. It has been hypothesized that this may be due to earlier architectural changes in ganglion cell bodies compared to their corresponding axons [50]. Prior papers have reported mixed data on the presence of attenuation of the pRNFL in HD patients [10, 11, 40, 51–54]. Most studies that found this attenuation may be specific to either the temporal or superior quadrant [11, 40, 51, 53] with only one study reporting significant differences in average pRNFL thickness [51]. Additionally, it has been shown that pRNFL attenuation may correlate with the degree of brain atrophy [55]. This could suggest that pRNFL attenuation is a later marker of disease progression when brain atrophy is more prevalent, while GCIPL attenuation occurs earlier, making it more relevant for biomarker development. Furthermore, when comparing GCIPL and pRNFL thicknesses in other neurodegenerative diseases, studies have observed increased sensitivity of GCIPL thickness for both disease diagnosis and severity [46, 48]. These findings suggest that GCIPL attenuation could be further investigated as a relevant biomarker for HD.

This study found no difference in CVI measurements between the HD and control groups. This is the first study investigating CVI in the eyes of HD participants, however, 2 prior studies have found decreased central subfoveal choroidal thickness (SFCT) [10, 52]. While both SFCT and CVI serve to characterize the choroid, there are some differences to note. SFCT can fluctuate significantly and can be influenced by age, axial length, and intraocular pressure of the eye at the time of imaging, in contrast to CVI which does not appear to respond to such variables [17]. Additionally, when comparing variability, including diurnal variation between SFCT and CVI, it was demonstrated that CVI was significantly less variable [17]. Given this, it has been suggested that CVI may be a more robust marker of choroidal vascularity than SFCT [17].

When interpreting our results, there are some limitations to consider. Control participants did not undergo genetic testing to ensure that they were gene negative for HD. However, they underwent an evaluation of family medical history with explicit questioning on the presence of any and all neurodegenerative disorders including HD. Given the autosomal dominant nature of the disease and its low national prevalence, individuals with no family history of HD are highly unlikely to test positive for mHtt [2]. Visual field testing and intraocular pressure measurements were not done to screen for glaucoma. Subjects instead were excluded through patient reported history, medical record review, visual acuity, and imaging review including both nonmydriatic ultra-widefield scanning laser ophthalmoscopy imaging (Optos California, Optos, Marlborough, MA) and RNFL quadrant thickness. We were not able to image patients with advanced HD; the limited cognitive and motor skills (head tremor/ chorea) of individuals with advanced HD patients hinders adequate fixation during imaging, ability to follow imaging cues provided by study staff, and proper positioning. There was significant variability in the number of images analyzed for the various retinal parameters measured. All images were graded by a masked author (D.S.G.) to assess for adequate image quality. There were more OCTA images excluded due to the greater susceptibility of OCTA images to motion artifact compared to OCT [56]. Our study did not analyze the deep capillary plexus as these images are often subject to projection artifact from overlying vessels in the superficial plexus and the overall yield of good quality deep plexus images is lower [57]. Additionally, our study analyzed pRNFL and did not assess macular RNFL which will be investigated in future work. Similarly, we did not analyze all of the individual layers of the neurosensory retina, because the software used in this study, which is a commercially available version, is currently unable to segment each of the layers. It should be noted that prior work has found significant attenuation of the outer retina (macular external limiting membrane–Bruch’s membrane complex) in both prodromal and motor manifest HD compared to matched controls [58]. In an effort to minimize potential image scaling errors caused by varying axial lengths [59], we excluded individuals with SEQ < -6D or > +6D. Prior work has found that this limits the magnitude of differences seen in OCTA parameters due to variations in axial length [60]. Finally, it is difficult to draw translational conclusions that change clinical care given the cross-sectional nature of our study and limited sample size; therefore, future studies incorporating longitudinal data in larger cohorts are needed to further ascertain the validity of using FAZ and GCIPL thickness as retinal HD biomarkers.

## Conclusion

We found decreased FAZ area in the SCP and decreased average GCIPL thickness in eyes of patients with gene-positive symptomatic HD compared to eyes of healthy control participants with no family history of HD. While choroidal structural parameters did not significantly differ between groups in this study, further work with larger patient populations may reveal subtle changes that may also be useful in differentiating these diagnostic groups. These findings suggest that further work to assess retinal imaging metrics as potential clinically relevant biomarkers for HD progression, stratification, and treatment response may be indicated.

## Supporting information

S1 Checklist STROBE statement—checklist of items that should be included in reports of observational studies. (DOCX)

S1 Table Correlation coefficients between retinal parameters and number of CAG repeats in the HD group. (DOCX)

S1 Data (XLSX)
