# Supplementary material for: Characterizing differences in retinal and choroidal microvasculature and structure in individuals with Huntington’s Disease compared to healthy controls: A cross-sectional prospective study
Source: PLoS One. 2024 Jan 30;19(1):e0296742. doi: 10.1371/journal.pone.0296742 (PMC10826956; doi:10.1371/journal.pone.0296742)
Supplement: S1 Checklist — (DOCX) [file pone.0296742.s001.docx]

STROBE Statement—checklist of items that should be included in reports of observational studies

|  | Item No. | Recommendation | Page  No. | Relevant text from manuscript |
| --- | --- | --- | --- | --- |
| **Title and abstract** | 1 | (*a*) Indicate the study’s design with a commonly used term in the title or the abstract | 1 | “cross-sectional prospective study” (title) |
|  |  | (*b*) Provide in the abstract an informative and balanced summary of what was done and what was found | 2 | “Forty-four eyes of 23 patients in the HD group and 77 eyes of 38 patients in the control group were analyzed. Average GC-IPL thickness and FAZ area were decreased in the HD group compared to controls (p = 0.015, p = 0.001). No other imaging metrics were significantly different between groups.” |
| Introduction | | | |  |
| Background/rationale | 2 | Explain the scientific background and rationale for the investigation being reported | 4 | “However, little is known about retinal manifestations in eyes of individuals with HD. Prior studies have found attenuation of various structural layers including the peripapillary retinal nerve fiber layer (RNFL), ganglion cell layer, and subfoveal choroid. (10, 11) However, changes in choroidal vascularity index (CVI) and retinal microvascular density are, as of yet, uncharacterized.” |
| Objectives | 3 | State specific objectives, including any prespecified hypotheses 4-5 |  | In this study, we used optical coherence tomography (OCT) and OCT angiography (OCTA) to characterize retinal and choroidal microvascular and structural changes in eyes of individuals who are gene-positive for mHtt and exhibit symptoms suggestive of HD compared to controls with normal cognition and no family history of HD. |
| Methods | | | |  |
| Study design | 4 | Present key elements of study design early in the paper | 5 | This prospective cross-sectional study was approved by the Duke Health Institutional Review Board (Pro00082598). |
| Setting | 5 | Describe the setting, locations, and relevant dates, including periods of recruitment, exposure, follow-up, and data collection | 5-6 | “Patients were seen at Duke Neurological Disorders Clinic” “Participants were recruited from Duke Neurological Disorders Clinic, the surrounding community, and the Duke Alzheimer’s Disease Prevention Registry of research volunteers with normal cognition.” “Participants were recruited from January 2019 to January 2023.” |
| Participants | 6 | (*a*) *Cohort study*—Give the eligibility criteria, and the sources and methods of selection of participants. Describe methods of follow-up  *Case-control study*—Give the eligibility criteria, and the sources and methods of case ascertainment and control selection. Give the rationale for the choice of cases and controls  *Cross-sectional study*—Give the eligibility criteria, and the sources and methods of selection of participants | 5-7 | See *Study Participants* section in methods (pages 5-7) |
|  |  | (*b*) *Cohort study*—For matched studies, give matching criteria and number of exposed and unexposed  *Case-control study*—For matched studies, give matching criteria and the number of controls per case | N/A |  |
| Variables | 7 | Clearly define all outcomes, exposures, predictors, potential confounders, and effect modifiers. Give diagnostic criteria, if applicable | 7-8 | See *OCTA and OCT Image Acquisition and Protocols* section in methods |
| Data sources/ measurement | 8* | For each variable of interest, give sources of data and details of methods of assessment (measurement). Describe comparability of assessment methods if there is more than one group | 7-8 | See *OCTA and OCT Image Acquisition and Protocols* section in methods |
| Bias | 9 | Describe any efforts to address potential sources of bias | 8 | Trained study staff manually assessed the quality of each image at the time of data collection. Image quality was subsequently reviewed by masked study staff prior to data analysis. Those images with poor scan quality (less than 7/10 signal strength index or significant image artifact) were excluded from statistical analysis. |
| Study size | 10 | Explain how the study size was arrived at | 5 | Participants were recruited from January 2019 to January 2023. Study size included all patients who were able to be enrolled during this time period. |

Continued on next page

| Quantitative variables | 11 | Explain how quantitative variables were handled in the analyses. If applicable, describe which groupings were chosen and why | 9 | Fisher’s exact test of differences between proportions and the Wilcoxon rank sum test were used for categorical and continuous variables, respectively |
| --- | --- | --- | --- | --- |
| Statistical methods | 12 | (*a*) Describe all statistical methods, including those used to control for confounding | 9 | To assess for possible confounders, demographic characteristics were compared. Fisher’s exact test of differences between proportions and the Wilcoxon rank sum test were used for categorical and continuous variables, respectively. Imaging parameters were compared with generalized estimating equations (GEE) in which age and sex were controlled for as covariates. GEE models were used to account for the correlation between 2 eyes of the same study participant. A Spearman’s rank correlation coefficient analysis was used to assess the relationship between the number of CAG repeats in a patient and their retinal imaging parameters. A Bonferroni correction was used to limit the increased error rate given the multiple comparisons used in the study. As such a p-value of < 0.002 was considered statistically significant. |
|  |  | (*b*) Describe any methods used to examine subgroups and interactions | 9 | Imaging metrics from the HD group were compared to the control group. SAS/STAT software, Version 9.4 of the SAS System for Windows (2002-2012 SAS Institute Inc.) was used to complete all statistical analyses. |
|  |  | (*c*) Explain how missing data were addressed | 8 | Those images with poor scan quality (less than 7/10 signal strength index or significant image artifact) were excluded from statistical analysis. |
|  |  | (*d*) *Cohort study*—If applicable, explain how loss to follow-up was addressed  *Case-control study*—If applicable, explain how matching of cases and controls was addressed  *Cross-sectional study*—If applicable, describe analytical methods taking account of sampling strategy |  | N/A |
|  |  | (*e*) Describe any sensitivity analyses |  | N/A |
| Results | | | | |
| Participants | 13* | (a) Report numbers of individuals at each stage of study—eg numbers potentially eligible, examined for eligibility, confirmed eligible, included in the study, completing follow-up, and analysed | 9-10 | “A total of 54 eyes from 28 participants in the HD group were imaged.” Forty-four eyes of 23 participants in the HD group (mean [SD] age, 54.9 [14.6]; 10 men [43.5%]), and 77 eyes of 39 patients in the control group (mean [SD] age, 56.9 [15.0]; 19 men [50.0%]) were analyzed. Of those in the HD group, 36 eyes of 19 patients were motor manifest HD and 8 eyes of 4 patients were prodromal HD.  In the control group, a total of 77 eyes of 39 patients were imaged. See sentence above for number of controls analyzed. |
|  |  | (b) Give reasons for non-participation at each stage | 9 | Of these 8 eyes, from 4 participants were excluded because they were asymptomatic for any of the motor, cognitive, or behavioral signs of motor manifest or prodromal HD. Two eyes from 1 patient were excluded due to a diagnosis of diabetes. Two eyes from 2 participants were excluded due to retinal detachment. Of these, 1 eye from 1 patient was excluded due to a Snellen visual acutity lower than 20/40. (control cohort) |
|  |  | (c) Consider use of a flow diagram |  | N/A |
| Descriptive data | 14* | (a) Give characteristics of study participants (eg demographic, clinical, social) and information on exposures and potential confounders | 10 | See *Table 1. Demographics* in results section |
|  |  | (b) Indicate number of participants with missing data for each variable of interest | 10-15 | See N row in *Tables 2-5* |
|  |  | (c) *Cohort study*—Summarise follow-up time (eg, average and total amount) |  | N/A |
| Outcome data | 15* | *Cohort study*—Report numbers of outcome events or summary measures over time |  | N/A |
|  |  | *Case-control study—*Report numbers in each exposure category, or summary measures of exposure |  | N/A |
|  |  | *Cross-sectional study—*Report numbers of outcome events or summary measures | 10-15 | See mean (SD) and Min, Median, Max row in *Tables 2-5* |
| Main results | 16 | (*a*) Give unadjusted estimates and, if applicable, confounder-adjusted estimates and their precision (eg, 95% confidence interval). Make clear which confounders were adjusted for and why they were included | 10-15 | See Min, Median, Max row in *Tables 2-5* |
|  |  | (*b*) Report category boundaries when continuous variables were categorized |  | N/A |
|  |  | (*c*) If relevant, consider translating estimates of relative risk into absolute risk for a meaningful time period |  | N/A |

Continued on next page

| Other analyses | 17 | Report other analyses done—eg analyses of subgroups and interactions, and sensitivity analyses | 13-16, Supplementary table 1 | A secondary analysis was performed comparing only motor manifest HD patients (rather than pooled motor manifest and prodromal HD patients as in the primary analysis) to the control group (Tables 4 and 5).  A Spearman’s rank correlation coefficient analysis was conducted to assess the relationship between the number of CAG repeats and the measured retinal parameters (Supplementary Table 1). The associations between CAG repeats and the analyzed retinal imaging parameters were not statistically significant. |
| --- | --- | --- | --- | --- |
| Discussion | | | | |
| Key results | 18 | Summarise key results with reference to study objectives | 16 | “This cross-sectional study is the first to identify decreased FAZ area in the SCP using OCTA in the eyes of individuals positive for mHtt and symptomatic for HD.” “In addition to the decreased FAZ area, we found significantly attenuated GC-IPL thickness in the HD group compared to control group. CVI did not significantly differ between groups.” |
| Limitations | 19 | Discuss limitations of the study, taking into account sources of potential bias or imprecision. Discuss both direction and magnitude of any potential bias | 20-21 | When interpreting our results, there are some limitations to consider. Control participants did not undergo genetic testing to ensure that they were gene negative for HD. However, they underwent an evaluation of family medical history with explicit questioning on the presence of any and all neurodegenerative disorders including HD. Given the autosomal dominant nature of the disease and its low national prevalence, individuals with no family history of HD are highly unlikely to test positive for mHtt.[2] Visual field testing and intraocular pressure measurements were not done to screen for glaucoma. Subjects instead were excluded through patient reported history, medical record review, visual acuity, and imaging review including both nonmydriatic ultra-widefield scanning laser ophthalmoscopy imaging (Optos California, Optos, Marlborough, MA) and RNFL quadrant thickness. We were not able to image patients with advanced HD; the limited cognitive and motor skills (head tremor/ chorea) of individuals with advanced HD patients hinders adequate fixation during imaging, ability to follow imaging cues provided by study staff, and proper positioning. There was significant variability in the number of images analyzed for the various retinal parameters measured. All images were graded by a masked author (D.S.G.) to assess for adequate image quality. There were more OCTA images excluded due to the greater susceptibility of OCTA images to motion artifact compared to OCT.[56] Our study did not analyze the deep capillary plexus as these images are often subject to projection artifact from overlying vessels in the superficial plexus and the overall yield of good quality deep plexus images is lower.[57] Additionally, our study analyzed pRNFL and did not assess macular RNFL which will be investigated in future work. Similarly, we did not analyze all of the individual layers of the neurosensory retina, because the software used in this study, which is a commercially available version, is currently unable to segment each of the layers. It should be noted that prior work has found significant attenuation of the outer retina (macular external limiting membrane – Bruch’s membrane complex) in both prodromal and motor manifest HD compared to matched controls.[58] In an effort to minimize potential image scaling errors caused by varying axial lengths,[59] we excluded individuals with SEQ < -6D or > +6D. Prior work has found that this limits the magnitude of differences seen in OCTA parameters due to variations in axial length.[60] Finally, it is difficult to draw translational conclusions that change clinical care given the cross-sectional nature of our study and limited sample size; therefore, future studies incorporating longitudinal data in larger cohorts are needed to further ascertain the validity of using FAZ and GCIPL thickness as retinal HD biomarkers. |
| Interpretation | 20 | Give a cautious overall interpretation of results considering objectives, limitations, multiplicity of analyses, results from similar studies, and other relevant evidence | 16-19 | See first 5 paragraphs of discussion |
| Generalisability | 21 | Discuss the generalisability (external validity) of the study results | 18,19 | “Since no prior studies on HD eyes have evaluated FAZ area, the clinical significance of this finding is uncertain.”  “These findings suggest that GC-IPL attenuation could be further investigated as a relevant biomarker for HD.” |
| Other information | |  | | |
| Funding | 22 | Give the source of funding and the role of the funders for the present study and, if applicable, for the original study on which the present article is based | n/a | The authors received no specific funding for this work. |

*Give information separately for cases and controls in case-control studies and, if applicable, for exposed and unexposed groups in cohort and cross-sectional studies.

**Note:** An Explanation and Elaboration article discusses each checklist item and gives methodological background and published examples of transparent reporting. The STROBE checklist is best used in conjunction with this article (freely available on the Web sites of PLoS Medicine at http://www.plosmedicine.org/, Annals of Internal Medicine at http://www.annals.org/, and Epidemiology at http://www.epidem.com/). Information on the STROBE Initiative is available at www.strobe-statement.org.
