# Supplementary material for: Characterizing differences in retinal and choroidal microvasculature and structure in individuals with Huntington’s Disease compared to healthy controls: A cross-sectional prospective study
Source: PLoS One. 2024 Jan 30;19(1):e0296742. doi: 10.1371/journal.pone.0296742 (PMC10826956; doi:10.1371/journal.pone.0296742)
Supplement: S1 Table — (DOCX) [file pone.0296742.s002.docx]

**Supplementary Table 1. Correlation coefficients between retinal parameters and number of CAG repeats in the HD group**

| **Parameters** | | **Number** | **Correlation** | **P-value*** |
| --- | --- | --- | --- | --- |
| OCTA | | | | |
|  | Perfusion density (3x3mm) | 36 | -0.104 | 0.548 |
|  | Perfusion density in ring (3x3mm) | 36 | -0.096 | 0.576 |
|  | Vessel density (3x3mm) | 36 | -0.008 | 0.963 |
|  | Vessel density in ring (3x3mm) | 36 | -0.041 | 0.812 |
|  | Perfusion density (6x6mm) | 36 | -0.108 | 0.530 |
|  | Perfusion density inner ring (6x6mm) | 36 | -0.226 | 0.186 |
|  | Perfusion density outer ring (6x6mm) | 36 | -0.083 | 0.631 |
|  | Vessel density (6x6mm) | 36 | -0.170 | 0.333 |
|  | Vessel density inner ring (6x6mm) | 36 | -0.217 | 0.203 |
|  | Vessel density outer ring (6x6mm) | 36 | -0.123 | 0.474 |
|  | Average capillary perfusion density | 36 | 0.023 | 0.900 |
|  | Average capillary flux index | 36 | 0.335 | 0.046 |
|  | FAZ area | 33 | -0.190 | 0.290 |
| OCT | | | | |
|  | Central subfield thickness | 41 | -0.098 | 0.543 |
|  | Average peripapillary retinal nerve fiber layer thickness (pRNFL) | 36 | 0.164 | 0.340 |
|  | pRNFL thickness - superior quadrant | 36 | 0.352 | 0.035 |
|  | pRNFL thickness - nasal quadrant | 36 | -0.182 | 0.288 |
|  | pRNFL thickness - inferior quadrant | 36 | 0.051 | 0.766 |
|  | pRNFL thickness - temporal quadrant | 36 | -0.015 | 0.932 |
|  | Average ganglion cell-inner plexiform layer thickness | 38 | -0.071 | 0.673 |
|  | CVI | 35 | 0.120 | 0.493 |
| *** p-value of < 0.002 was considered statistically significant  *CAG: cytosine-adenine-guanine*  *HD: Huntington’s disease*  *OCTA: optical coherence tomography angiography*  *FAZ: foveal avascular zone*  *OCT: optical coherence tomography*  *CVI: choroidal vascularity index* | | | | |
